# HLA genotyping by next-generation sequencing of complementary DNA

**DOI:** 10.1186/s12864-017-4300-7

**Published:** 2017-11-28

**Authors:** Hidenobu Segawa, Yoji Kukita, Kikuya Kato

**Affiliations:** 1 Department of Molecular and Medical Genetics, Research Institute, Osaka Medical Center for Cancer and Cardiovascular Diseases, 1-3-3 Nakamichi, Higashinari-ku, Osaka, 537-8511 Japan; 2 Laboratory of Medical Genomics, Nara Institute of Science and Technology, 8916-5 Takayama, Ikoma, Nara, 630-0101 Japan

**Keywords:** Next-generation sequencing, cDNA, HLA, Molecular barcode

## Abstract

**Background:**

Genotyping of the human leucocyte antigen (HLA) is indispensable for various medical treatments. However, unambiguous genotyping is technically challenging due to high polymorphism of the corresponding genomic region. Next-generation sequencing is changing the landscape of genotyping. In addition to high throughput of data, its additional advantage is that DNA templates are derived from single molecules, which is a strong merit for the phasing problem. Although most currently developed technologies use genomic DNA, use of cDNA could enable genotyping with reduced costs in data production and analysis. We thus developed an HLA genotyping system based on next-generation sequencing of cDNA.

**Methods:**

Each HLA gene was divided into 3 or 4 target regions subjected to PCR amplification and subsequent sequencing with Ion Torrent PGM. The sequence data were then subjected to an automated analysis. The principle of the analysis was to construct candidate sequences generated from all possible combinations of variable bases and arrange them in decreasing order of the number of reads. Upon collecting candidate sequences from all target regions, 2 haplotypes were usually assigned. Cases not assigned 2 haplotypes were forwarded to 4 additional processes: selection of candidate sequences applying more stringent criteria, removal of artificial haplotypes, selection of candidate sequences with a relaxed threshold for sequence matching, and countermeasure for incomplete sequences in the HLA database.

**Results:**

The genotyping system was evaluated using 30 samples; the overall accuracy was 97.0% at the field 3 level and 98.3% at the G group level. With one sample, genotyping of DPB1 was not completed due to short read size. We then developed a method for complete sequencing of individual molecules of the DPB1 gene, using the molecular barcode technology.

**Conclusion:**

The performance of the automatic genotyping system was comparable to that of systems developed in previous studies. Thus, next-generation sequencing of cDNA is a viable option for HLA genotyping.

**Electronic supplementary material:**

The online version of this article (10.1186/s12864-017-4300-7) contains supplementary material, which is available to authorized users.

## Background

Genotyping of the human leucocyte antigen (HLA) is indispensable for transplantation of hematopoietic stem cells or organs [1–3] and for the management of other diseases involving immune reactions [4]. However, unambiguous genotyping is technically challenging due to high polymorphism of the corresponding genomic region [5, 6]. Currently, polymerase chain reaction sequence-specific oligonucleotide probe (PCR-SSOP) [7, 8] such as Luminex Technology [9, 10] and sequence-based typing (SBT) by the Sanger method [11, 12] are the standards for HLA typing. However, both approaches have difficulties resulting from chromosomal phase (*cis/trans*) ambiguity.

G group typing, namely, identification of the amino acid sequences of antigen-binding regions (amino acid sequences of exons 2/3 for class I and exon 2 for class II), is the most crucial for successful transplantation, with an identical G group type being required between the donor and recipient. Several studies have reported a better transplantation success rate with field 2 typing (identification of the amino acid sequences of coding regions) than with G group typing [13–15]. Because the expression of HLA genes has been reported to affect hematopoietic stem cell transplantation [16], the detection of null alleles (alleles for which no HLA products are expressed at the cell surface) may be important. Detection of null alleles typically needs field 3 typing (identifying the nucleotide sequences of coding regions), though some null alleles can only be detected by field 4 typing (nucleotide sequencing of the entire gene, including introns and untranslated regions). Field 3/4 typing is also useful to identify associations between diseases such as HLA-B*57:01 and Abacavir hypersensitivity [17]. There were polymorphisms affecting gene expression levels in the promoter regions of HLA genes, suggesting the potential merit of field 4 typing [18–20]. These points should be carefully evaluated when choosing the typing method.

Introduction of massively parallel DNA sequencing, namely next-generation sequencing, is changing the landscape of typing. In addition to the high throughput of data, the additional advantage of massively parallel sequencing is that DNA templates are derived from single molecules. This is a strong merit for the phasing problem. There are a number of methods using massively parallel sequencers such as Roche FLX [21, 22], MiSeq [23–25], and Ion Torrent PGM [26, 27]. Most of the studies employ PCR for target enrichment, but hybridization capture is also used [28]. Combinations with long-range PCR covering large target regions could potentially perform field 4 level of genotyping.

Instead of long-range PCR for target genomic regions, sequencing of RNA, namely complementary DNA, could be an effective alternative. Because DNA sequences of introns and untranslated regions are not indispensable to deduce protein sequences, the field 3 level is sufficient for HLA typing. Compared with genome sequencing, cDNA sequencing reduces the costs for both data production and analysis, and could be a viable approach for HLA typing [29–31]. In genome sequencing, the sequence information of introns and untranslated regions can be utilized for the resolution of phasing ambiguity. In cDNA sequencing, resolution of phasing ambiguity without information intrinsic to genome sequences is often not straightforward because polymorphic regions are often not covered within a single read due to the short read size in next-generation sequencing.

We developed an HLA genotyping system based on cDNA sequencing using Ion Torrent PGM. The data analysis system exhibited an automatic typing ability comparable to that of previous methods based on genome sequencing. In addition, for rare cases where conventional sequencing could not assign haplotypes, a template preparation and sequencing method using molecular barcodes was introduced.

## Results

### Preparation of templates and next-generation sequencing

The HLA genotyping system 01 includes generation of sequence data using conventional template preparation and subsequent data analysis to assign HLA types. cDNA was synthesized with random primers to cover the entire region of each HLA gene. For PCR amplification, HLA class I and class II genes were divided into 4 and 3 parts, respectively. Each part was subjected to sequencing from 2 directions, i.e., forward and reverse, and consequently, HLA class I and class II genes were divided into 8 and 6 target regions, respectively. In total, 27 pairs of PCR primers were designed. The primer sequences are shown in Additional file 1: Table S1. Adapters including sequences for indexing samples and sequencing with Ion Torrent PGM were then attached to the PCR products. After the template preparation step, sequencing was performed with Ion Torrent PGM. The details of the experimental process are described in the Materials and Methods section.

### Outline of data analysis in the HLA typing system 01

**Fig. 1:**
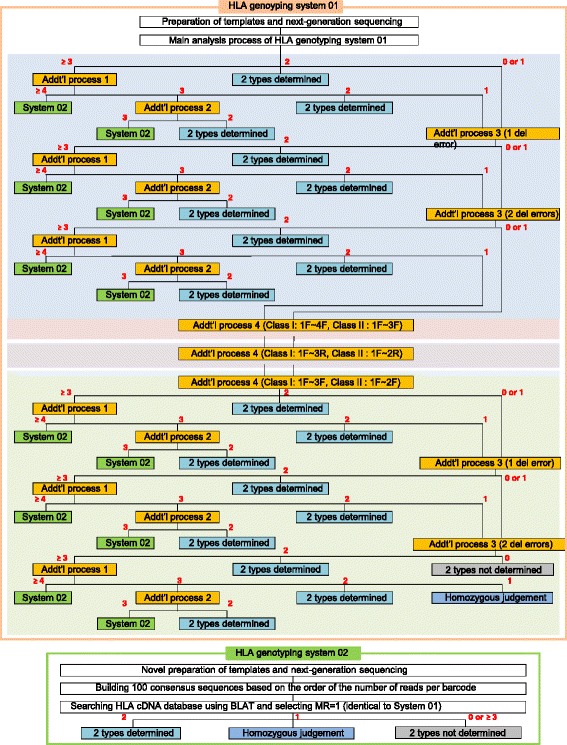
Schematic representation of the HLA typing system. The HLA genotyping system 01 was the main system, which included 4 additional analysis processes. HLA genotyping system 02 was based on the complete sequencing of individual molecules with molecular barcodes, and only for the DPB1 alleles that system 01 could not finish typing

The output sequences were subjected to the data analysis system of the HLA genotyping system 01. A graphical representation of the entire system is shown in Fig. 1. This system is designed to perform automatic HLA typing, and includes the main analysis process and 4 additional analysis processes for cases where the main process could not complete typing. The principle of the main process is constructing candidate sequences generated from all possible combinations of variable bases and arranging them in decreasing order of the number of reads. Development and optimization of the system was done using 12 RNA samples: 3 purified from peripheral blood, and 9 from breast cancer tissues.

### The main process of the HLA genotyping system 01

The main process consists of the following 3 steps: a) building DNA sequences for analysis, b) listing candidate sequences, and c) assignment of HLA types.

- Building DNA sequences for analysis
  The output sequences were classified as HLA class I (HLA-A, −B, −C) or class II(HLA-DRB1/3/4/5, −DQB1, −DQA1, −DPB1)genes. After removing the primer sequences, the system collected the initial 55% or 60% sequences of the target regions from each sequence read. The fraction of recovery was determined with each target region. This process is indispensable because sequencing errors are frequent in the removed regions.
- Selection of candidate sequences of target regions
  First, all sequences of a target region were aligned using the multiple alignment software, multiple sequence comparison by log-expectation (MUSCLE, http://www.drive5.com/muscle) [32]. Next, all base positions with 2 or more bases were listed. Thirdly, sequences generated from all combinations of variable bases were listed with the number of reads. These sequences were candidates of haplotypes, and the number of reads was termed as haplotype score. Variable bases might occur due to haplotypes or due to experimental errors. Sequences whose haplotype score was less than 1/*m* of the highest haplotype score were removed from the candidate sequences, because their variable bases were possibly due to experimental errors. *m* is a parameter set for each HLA gene. Determination of *m* is described later along with another parameter, *n*.
- Assignment of HLA types
  Candidate sequences were searched in the HLA cDNA database (http://www.ebi.ac.uk/ipd/imgt/hla/) [33] for HLA types using the BLAST-like alignment tool (BLAT, http://genome.ucsc.edu/). BLAT only reports substitutions and insertions/deletions in continuous matched sequences, and cannot detect a stretch of unmatched terminal sequences. Examples are shown in Additional file 2: Figure S1. We therefore introduced a parameter named matching rate (MR) representing the ratio of matched bases to total bases. HLA types corresponding to candidate sequences with MR = 1 were selected. The results of all target regions, 8 each for HLA class I gene and 6 each for HLA class II gene, were collected and the HLA types of the 2 alleles were determined.

In the majority of cases, the results of target regions did not contradict each other, and were assigned 2 types. Cases for which the main process did not assign 2 haplotypes were subjected to the following additional analysis processes as shown in the flowchart in Fig. 1.

### Additional analysis processes for genes with more than 2 haplotypes

These cases included at least one wrongly assigned HLA type. The following analysis processes, designated as additional processes 1 and 2, were developed to remove the wrong HLA types.

Additional process 1: Selection of candidate sequences applying more stringent criteria.

In some cases, removal of sequences with experimental errors in the main process was not sufficient. Candidate sequences had errors accidentally matching the HLA types in the database. In such cases, more stringent criteria were applied for the selection through haplotype score: HLA types containing target regions whose haplotype scores are less than 1/*n* of the highest haplotype score were removed, and then assigned HLA types. *n* is a parameter set for each HLA gene, and is smaller than another parameter, *m*, in the main process. The optimum pairs of parameters, *m* and *n*, were determined using the learning data set and are shown in Table 1.

**Table 1:**
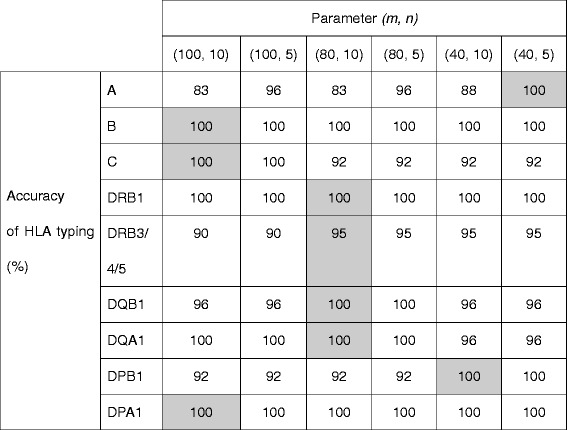
Parameters *m* and *n* as thresholds for haplotype scores. Values of *m* and *n* in corresponding shaded cells were selected

Additional process 2: Removal of artificial haplotypes.

**Fig. 2:**
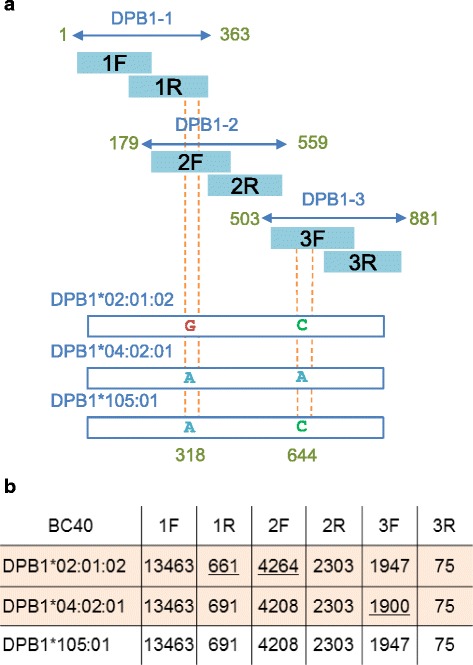
A case with an artifactual candidate sequence generated from the sequences of 2 haplotypes. **a**, schematic representation of the case. **b**, haplotype scores of candidate sequences. The sequence corresponding to DPB1*105:01 is an artifactual sequence generated from the above 2 haplotypes

In our strategy, the entire coding region of each gene was generated from 8 or 6 target regions. Artificial haplotypes combining the sequences of different alleles may therefore be selected. An example is shown in Fig. 2. In this case, variable bases were located in 2 target regions (2F and 3F) (Fig. 2a). Because the chance that different sequences have the same haplotype score is extremely low, haplotype score can be used as an identifier of individual sequences. The third HLA type, DPB1*105:01, had haplotype scores from both the first and the second haplotypes (Fig. 2b). Haplotype scores of the first and second HLA types did not share any of the haplotype scores. The third HLA type was therefore concluded as an artificial haplotype generated from the first and second.

### Additional analysis processes for genes with less than 2 candidates

These cases included those with a homozygous haplotype and those for which at least 1 type not identified. The following additional analysis processes were developed.

Additional process 3: Selection of candidate sequences with a relaxed threshold of MR.

Insertion/deletion errors are frequent in Ion Torrent sequencers. In some cases, deletion errors at particular positions prevented the identification of candidate sequences. To resolve this situation, selection of candidate sequences was performed again under a relaxed criterion, namely MR ≥ 0.99, allowing 1 and then 2 deletion errors.

Additional process 4: Countermeasure for incomplete sequences in the HLA database.

In the HLA database, there are entries lacking sequences near the 3′ end, because only the antigen-binding region (exons 2 and 3 for class I and exon 2 for class II) is required for registration. In such cases, haplotype scores in the target region(s) near the 3′ end were lacking. To accommodate this situation, the final step in the assignment of HLA types was performed by removing a target region from the 3′ end successively. This process was repeated up to 3 times. If 2 haplotypes were still not assigned, the case was considered homozygous.

### Performance of the HLA typing system 01

**Fig. 3:**
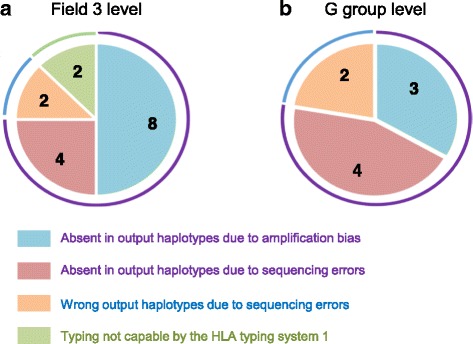
The causes of failed typing. **a**, field 3 level HLA typing. **b**, G group level HLA typing

We tested the performance of the HLA typing system 01 using 30 new samples purified from colorectal and breast cancer tissues. Typing results with cloning and Sanger sequencing were used as the reference. The accuracies of the system are shown in Table 2. The accuracies ranged from 95% to 100% both at the field 3 and G group levels. The overall accuracy was 97.0% at the field 3 level and 98.3% at the G group level. The causes of failed typing are summarized in Fig. 3. 82% of the alleles (between 45 and 60 of the 60 assessed for each gene) were determined using only the main process.

**Table 2:** Accuracy of the HLA typing system deduced from comparison with data generated by cloning and Sanger sequencing

	Total	A	B	C	DRB1	DRB 3/4/5	DQB1	DQA1	DPB1	DPA1
a. Field 3 level										
HLA typing system	516	58	57	57	59	51	57	59	58	60
Sanger sequencing	532	60	60	60	60	52	60	60	60	60
Accuracy (%)	97.0	96.7	95.0	95.0	98.3	98.1	95.0	98.3	96.7	100
b. G group level										
HLA typing system	523	58	57	59	59	51	60	59	60	60
Sanger sequencing	532	60	60	60	60	52	60	60	60	60
Accuracy (%)	98.3	96.7	96.7	95.0	98.3	98.1	100	98.3	100	100

Figures indicate the number of alleles

As there have been reports of HLA alleles with low expression levels, [34–37], the absence of certain alleles may be due to a bias in transcript numbers. However, because these cases did not include low expression alleles, we conclude that the cause of non-detection was an amplification bias.

The performance of our typing system was compared with that of automatic typing systems from 3 other representative studies. The comparison is shown in Additional file 3: Table S2. Notably, the data sets used for evaluation were not the same. Ozaki et al. reported an HLA typing system based on genome sequencing with Ion Torrent PGM [26]. The performance of this system was excellent with class I genes, but was poor with DRB1 and lacking with DQA1 and DPA1. Another typing system based on genome sequencing with Illumina MiSeq presented by Nelson et al. covered all the HLA genes and presented a constantly good performance [25]. The overall fraction of determined haplotypes was 95.4% with the system reported by Nelson et al. and was 93.7% with our system. The typing system presented by Lank et al. was based on cDNA sequencing with Roche GS junior [31]. This system only covered the DRB genes among class II genes, and its overall accuracy was 89.4%.

### HLA genotyping system 02: Complete sequencing of individual molecules with molecular barcodes

**Fig. 4:**
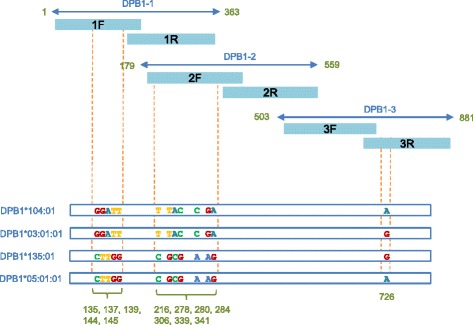
Example whose haplotypes cannot be determined with HLA typing system 01. Unlike the example shown in Fig. 2, 2 artifactual candidate sequences are generated in this case

**Fig. 5:**
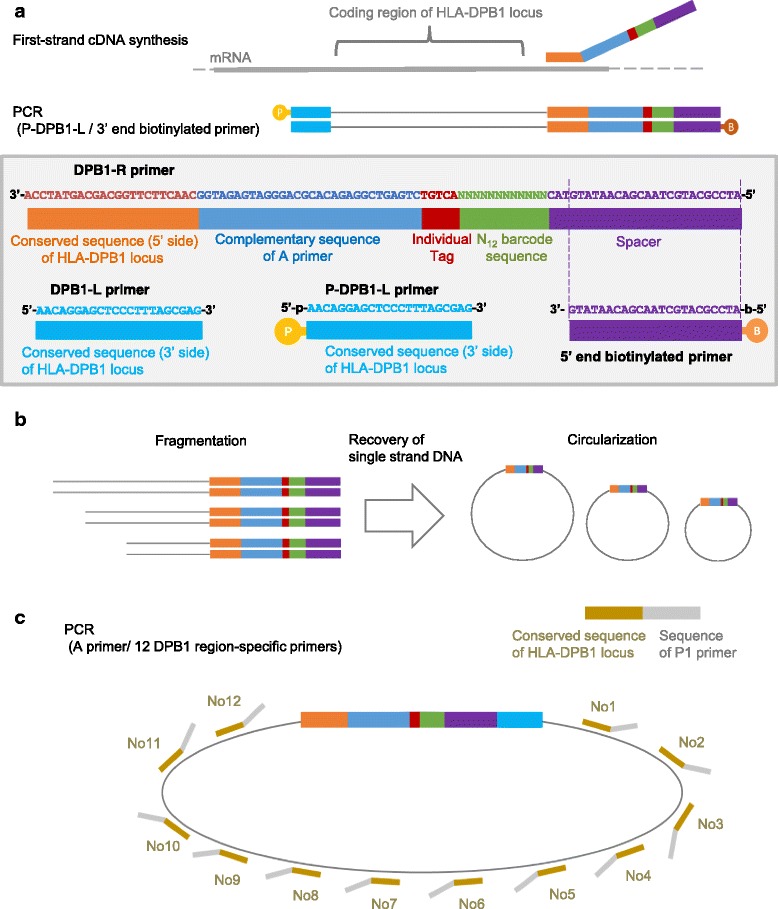
Complete sequencing of individual cDNA molecules with molecular barcodes. **a**, cDNA synthesis and PCR. **b**, Fragmentation and circularization. **c**, Template preparation through PCR using a circularized molecule as the template

The single failed case of DPB1 had more than 3 candidate HLA types due to variable bases in 3 target regions, and the additional process 1 could not determine 2 haplotypes. Schematic representation of this case is shown in Fig. 4. In such cases, the sequence data covering all variable base positions are indispensable for typing. We applied the molecular barcode technology for complete sequencing of individual cDNA molecules, and named this process as HLA genotyping system 02. The method was developed only for DPB1. A schematic representation of the method is shown in Fig. 5. The outline of the method is as follows.

1. cDNA was synthesized with the DPB1-R primer containing the 3′ end common sequence of DPB1 and a barcode-generating sequence, namely N_12_. PCR was then performed with the DPB1-L primer, P-DPB1-L primer, and the 5′ end biotinylated primer (Fig. 5a).
2. The PCR product was fragmented with dsDNA fragmentase, followed by the recovery of single strand DNA using streptavidin-coated beads, and circularization (Fig. 5b).
3. PCR amplification of the fragmented molecules was performed with a mixture of 12 DPB1 region-specific primers and the A primer, followed by sequencing from the cleaved ends (Fig. 5c).

Output sequences with the same barcode were grouped, and consensus sequences were constructed for each region-specific primer. In total, 100 consensus sequences were selected based on the order of the number of reads per barcode. The consensus sequences were then mapped onto the candidate sequences selected by the HLA typing system 01. For the case shown in Fig. 4, the results indicated DPB1*03:01:01 and DPB1*05:01:01 as the haplotypes.

## Discussion

HLA typing by next-generation sequencing generally uses genomic DNA as the template source. Because methods based on amplification of individual exons inevitably yield indeterminable haplotypes due to phasing ambiguity, template preparation with long-range PCR is indispensable for typing based on genome sequencing. The major merit of HLA typing based on cDNA sequencing is the short size of the sequencing target regions. The target size in cDNA sequencing is approximately one fifth that of the genomic regions. For example, the size of the entire target regions from genome sequencing of Ozeki et al. [26] was 46.2 kb, but the size of the corresponding region from our cDNA sequencing was 9.2 kb. One potential demerit is the loss of information from intron regions to identify haplotypes, especially for cases with phase ambiguity. Due to the short read size in next-generation sequencing, resolution of phasing ambiguity is a difficult problem. We solved this problem completely only by improving our data analysis system for most cases. Our data analysis system, the HLA typing system 01 alone, could not complete the level 4 typing with only a single DPB1 case. We therefore introduced complete sequencing of individual molecules with molecular barcodes for DPB1. This technique was originally developed for complete sequencing of individual molecules of viral genomes [38, 39]. Although this sequencing system with molecular barcodes removes all ambiguities in typing, its inclusion in routine typing should be considered carefully because it is labor-intensive and not indispensable for the majority of cases.

The major causes of failed typing were sequencing errors and bias during PCR. Both causes are general problems that are not intrinsic to HLA typing. Our HLA typing system uses multiple alignment software for the selection of candidate sequences of haplotypes. Sequence errors mainly affect this process, and different multiple alignment softwares may improve the performance. Bias during PCR may be removed or reduced by applying new primers and/or refined annealing conditions. Alleles with low expression levels [34] may also present a potential problem, which may be assessed in the next step of development.

## Conclusions

We demonstrated that next-generation sequencing of cDNA is a viable approach for HLA typing. Considering its cost-effectiveness, cDNA sequencing has enough chance to become the method of choice.

## Methods

### Samples

Human peripheral blood samples were obtained from three healthy male volunteers. Colorectal and breast cancer tissues were frozen and stored at −80 °C after surgery. Written informed consent was obtained from all patients. This study was approved by the ethics committee of Osaka Medical Center for Cancer and Cardiovascular Diseases.

### RNA extraction

Peripheral blood mononuclear cells (PBMC) were separated using Ficoll-Paque PLUS (GE Healthcare, Little Chalfont, Buckinghamshire, UK), according to the manufacturer’s instructions, except that we used phosphate buffered saline (PBS) instead of the “Balanced Salt Solution” described in the procedure. Total RNA was isolated from separated PBMCs using TRIzol reagent (Thermo Fisher Scientific, Waltham, MA, USA), according to the manufacturer’s instructions. Total RNA from colorectal and breast cancer tissues were extracted from 10 serial sections (20 μm) of frozen tissue, using TRIzol reagent.

### First-strand cDNA synthesis for the HLA typing system 01

Total RNA was adjusted to 20 ng/μl, and 100 ng was reverse transcribed using Super Script III Reverse Transcriptase (Thermo Fisher Scientific) with random hexamers, according to the manufacturer’s instructions.

### Primers

To amplify the coding regions of 11 HLA loci (HLA class I: HLA-A, -B, -C, HLA class II: HLA-DRB1/3/4/5, -DQB1, -DQA1, -DPB1, -DPA1), we designed specific primers in highly conserved regions, which were found by aligning sequences in the IMGT/HLA database by the multiple alignment tool, MUSCLE (http://www.drive5.com/muscle) shown in Additional file 1: Table S1.

### PCR and mixing PCR products in equal amounts

PCR was performed with the following reaction setup: 1 μl first-strand cDNA solution, 4 μl of 5× Q5 buffer (NEB, Ipswich, MA, USA), 1.6 μl of 2.5 mM dNTP, 2 μl of 5 μM primer pairs, 11.2 μl of Nuclease-Free water, and 0.2 μl of Q5 Hot Start High-Fidelity DNA Polymerase (NEB). The following cycling conditions were used: 1 cycle of 98 °C for 30 s, 30 cycles of 98 °C for 10 s, 50 °C for 10 s, and 72 °C for 30 s, and 1 cycle of 72 °C for 2 min.

The concentration of each PCR product was measured using the Quanti-iT™ dsDNA Assay Kit, Broad range (Thermo Fisher Scientific) according to the manufacturer’s instructions and the PCR products were mixed in equal amounts. Mixed PCR products were purified using the AMPure XP kit (Beckman Coulter, Brea, California, USA) according to the manufacturer’s instructions. The concentration of the purified mixed PCR products was measured using Qubit dsDNA HS Assay Kit (Thermo Fisher Scientific) according to the manufacturer’s instructions.

### Massively parallel sequencing

Barcoded libraries were prepared using Ion Xpress plus Fragment Library Kit (Thermo Fisher Scientific) and Ion Xpress barcode Adaptors 1-16 Kit (Thermo Fisher Scientific) according to the manufacturer’s instructions. Each barcoded library was mixed in equal amounts and diluted to 100 pM. Using 2.5 μl of the 100 pM mixed barcoded library, we prepared sequencing templates with Ion One Touch™ 2 (Thermo Fisher Scientific) and Ion One Touch™ ES (Thermo Fisher Scientific). Sequencing was performed using the Ion PGM™ Sequencing 400 Kit (Thermo Fisher Scientific) according to the manufacturer’s instructions for the 318v2 chip. Torrent Suite software (Thermo Fisher Scientific) was used to extract reads for individuals (BAM files). For each locus, one of the HLA sequences in RefSeq (https://www.ncbi.nlm.nih.gov/refseq/) was used as a reference sequence. BAM files were converted to SAM files using SAMTools [40].

### Building DNA sequences for analysis

We created two sequence files (sense and antisense directions) per amplified region. After removing the primer sequences, the initial 55% or 60% sequences of the target regions from each sequence read were recovered. When the number of reads was more than 10,000, we selected 10,000 reads randomly.

### Selection of candidate sequences of target regions

The sequences of each file were aligned by MUSCLE with the parameter setting ‘-maxiters 2’. If the fraction of a base at a base position were less than 1%, the base was regarded as a sequencing error and was removed. We removed the sequences with low haplotype scores using the thresholds described above.

### Assignment of HLA types

“Haplotype candidates” (Additional file 4: Figure S2) of each target region were aligned to the HLA cDNA database using BLAT [41] with the parameter setting ‘-minIdentity 100’. We calculated the “matching rate (MR)” using the number of mismatched bases from the BLAT result. The denominator of “MR” (Overlapped Length: OL in Additional file 4: Figure S2) which was the overlapped length between the query sequence (candidate sequence) and target sequence in the HLA cDNA database, was calculated using the following 9 factors in Additional file 4: Figure S2A: MSPQ (Mapped Start Position of Query; mapped start position of “Haplotype candidate” at reference sequence), MEPQ (Mapped End Position of Query; mapped end position of “Haplotype candidate” at reference sequence), Lq (Length of query; the length of “Haplotype candidate”), QS (Query Start; aligned start position of “Haplotype candidate”), Tgap (Target gap; the number of deletion bases in “Haplotype candidate”), MSPT (Mapped Start Position of Target; mapped start position of “HLA cDNA database” at reference sequence), MEPT (Mapped End Position of Target; mapped end position of “HLA cDNA database” at reference sequence), Lt (Length of target; the length of “HLA cDNA database”), TS (Target Start; aligned start position of “HLA cDNA database”). We calculated the MEPQ, MSPT, and MEPT as below.

MEPQ = MSPQ + Lq + Tgap −1.

MSPT = MSPQ + QS – TS.

MEPT = MSPT + Lt −1.

The OL (Overlapped Length) between the “Haplotype candidate” and “HLA cDNA database” was calculated as follows. A schematic representation is presented in Additional file 4: Figure S2B.

Case I: when MSPQ ≦ MSPT and MEPT ≦ MEPQ, OL = Lt.

Case II: when MSPQ ≦ MSPT and MEPT > MEPQ, OL = MEPQ – MSPT +1.

Case III: when MSPQ > MSPT and MEPT ≦ MEPQ, OL = MEPT – MSPQ +1.

Case IV: when MSPQ > MSPT and MEPT > MEPQ, OL = Lq + Tgap.

### First-strand cDNA synthesis and PCR for coding region of HLA-DPB1 locus for complete sequencing of individual molecules with molecular barcodes

Total RNA (100 ng) was reverse transcribed using Super Script III Reverse Transcriptase (Thermo Fisher Scientific) with the DPB1-R primer. First PCR for the coding region of HLA-DPB1 locus was performed with the following reaction including 1 μl of the first-strand cDNA solution, 16.99 μl of Nuclease-Free water, 2.5 μl of 10× High Fidelity PCR Buffer, 2.2 μl of 2.5 mM dNTP, 1.2 μl of 50 mM MgSO4 (Thermo Fisher Scientific), 0.11 μl of 5 unit/μl Platinum Taq, 0.5 μl of 10 μM DPB1-L, 0.5 μl of 10 μM 5′ end biotinylated primer. The following cycling conditions were used: 1 cycle of 94 °C for 30 s, 30 cycles of 94 °C for 30 s, 65 °C for 30 s, and 68 °C for 1 min. The second PCR was performed using the purified first PCR products and phosphorylated DPB1-L (P-DPB1-L) in 8 wells (total 160 μl). The cycling conditions were the same as those of the first PCR. Total PCR products were purified using AMPure XP. The primer sequences are shown in Fig. 5a.

### Fragmentation of biotinylated PCR products

For fragmentation of the biotinylated PCR products, a 20 μl solution containing 2.4 μl of 84.2 ng/μl purified biotinylated PCR products (200 ng), 2 μl of 10× Fragmentase Reaction Buffer v2 (NEB), 13.6 μl of Nuclease-Free water, and 2 μl dsDNA fragmentase (NEB) was incubated at 37 °C for 5 min, and 7.5 μl of 0.5 M EDTA was added to inactivate the enzyme. Fragmented PCR products were purified using AMPure XP (recovery volume: 20 μl).

### Preparation of single-strand DNA

Single-strand DNA was prepared from fragmented PCR products using Dynabeads® MyOne™ Streptavidin C1 (Thermo Fisher Scientific). Fragmented PCR products and 100 μg of beads washed using 1× B&W Buffer (5 mM pH 7.5 Tris-HCL, 0.5 mM EDTA, and 1 M NaCl) were mixed, stirred at intervals of 3 min, and incubated at 25 °C for 15 min in 20 μl of 2× B&W Buffer. After incubation, the mixture was placed on a magnetic bead separator for 3 min and the supernatants were removed. Beads-fragmented PCR products complexes were washed thrice in 1× B&W Buffer. Next, we performed alkaline denaturation to recover the single-stranded unbiotinylated products. After adding 100 μl of the alkaline solution (0.1 M NaOH and 1 mM EDTA) and 1 μl of 10% Triton X-100 to the above complexes, the mixture was stirred at intervals of 2 min and incubated at 25 °C for 10 min; the supernatants were then transferred to a 1.5 ml LoBind Tube. This process was repeated to recover the remaining single-stranded unbiotinylated products. In total, we recovered 200 μl of alkaline solution containing single-stranded unbiotinylated products. This solution was neutralized with 20 μl of 1 M HCL, and purified using the NEB Monarch PCR DNA clean up kit (NEB). The concentration of purified single-strand products was measured by Real-time PCR with DPB1-RT-F and DPB1-RT-R primer in Additional file 5: Figure S3. To prepare the calibration curve, we used pre-fragmented PCR products as templates.

### Circularization

For circularization, a 20-μl solution containing 2 μl of 10× T4 RNA Ligase Buffer (NEB), 0.4 μl of 10 mM ATP, 4 μl of 50% PEG8000 (NEB), 0.2 μl of T4 RNA Ligase 1 (NEB), 3.7 μl of Nuclease-Free water, and 10 μl of 20 amol/μl single-strand products was incubated at 37 °C for 16 h and 65 °C for 15 min to inactivate the enzyme. This reaction was performed in 8 tubes. In total, 240 μl of the circularized products were purified using the NEB Monarch PCR DNA clean up kit (NEB).

### PCR of circularized single-strand DNA and massively parallel sequencing

PCR was performed with the following reaction setup: 1 μl of purified and circularized DNA solution, 16.99 μl of Nuclease-Free water, 2.5 μl of 10× High Fidelity PCR Buffer, 2.2 μl of 2.5 mM dNTP, 1.2 μl of 50 mM MgSO4, 0.11 μl of 5 unit/μl Platinum Taq, 0.5 μl of 10 μM A primer, 0.5 μl of 10 μM P1-DPB1-No1~No12 in shown in Fig. 5c and Additional file 6: Table S3. The following cycling conditions were used: 1 cycle of 94 °C for 30 s, 40 cycles of 94 °C for 30 s, 65 °C for 30 s, and 68 °C for 1 min. After mixing each PCR product, gel extraction (range from 200 bp to 500 bp) was performed using the MinElute Gel Extraction Kit (Qiagen, Hilden, Germany). The concentration of the purified products was measured using the Quanti-iT™ dsDNA Assay Kit, Broad range. Using 2.5 μl of 100 pM purified products, we prepared sequencing templates with Ion One Touch™ 2 (Thermo Fisher Scientific) and Ion One Touch™ ES (Thermo Fisher Scientific). Sequencing was performed using the Ion PGM™ Sequencing Hi-Q Kit (Thermo Fisher Scientific).

### Data analysis for the complete sequencing of individual molecules with molecular barcodes

Reads with the same barcode tags were grouped together, and the number of reads per barcode tag was counted. Using groups whose number of reads per barcode tag was ranked within the top 100, consensus sequences were created for each group using the following 2 steps.

Step 1: Creating consensus sequences for reads generated with each region-specific primer, i.e., P1-DPB1-No1~No12.

Reads of each group (total 100 groups) were aligned to the reference sequence of HLA-DPB1 locus (NM_002121.5) with BWA (version 0.7.15) [42]. Consensus sequences were generated for each group of reads sharing the same mapping positions at both ends using IGVTools (https://software.broadinstitute.org/software/igv/igvtools) to reduce amplification bias and to remove PCR errors.

Step 2: Creating consensus sequences of the entire region.

Consensus sequences generated in step 1 were aligned to the reference sequence again, and a consensus sequence of the entire region was created.

One hundred consensus sequences from the entire region were subjected to the HLA typing system.

### Sanger sequencing

PCR was performed using primers designed outside the coding region for the HLA locus. PCR products were subcloned in *E coli*. The sequencing reaction was performed with Big Dye v3.1 (Thermo Fisher Scientific), and the sequences were determined by capillary electrophoresis using the ABI PRISM 3100 Genetic Analyzer (Thermo Fisher Scientific).

## Additional files

Additional file 1: Table S1. The sequence of the primers for HLA typing system 01. (XLS 30 kb)

Additional file 2: Figure S1. Examples of alignment with BLAT. (PPT 70 kb)

Additional file 3: Table S2. Comparison of the performance of automatic HLA typing. (XLS 28 kb)

Additional file 4: Figure S2. Calculation of the OL (Overlapped Length) between sequences of “Haplotype candidates” and “HLA cDNA database”. (PPT 174 kb)

Additional file 5: Figure S3. Primers for measuring single-strand products by Real-time PCR. (PPT 139 kb)

Additional file 6: Table S3. Primers for PCR of circularized single-strand DNA. (XLS 34 kb)

## Abbreviations

BLAT: BLAST-like alignment tool
HLA: Human leucocyte antigen
MR: Matching rate
MUSCLE: Multiple sequence comparison by log-expectation
PBMC: Peripheral blood mononuclear cells
PBS: Phosphate buffered saline
PCR-SSOP: Polymerase chain reaction sequence-specific oligonucleotide probe
SBT: Sequence-based typing
