# Supplementary material for: HLA genotyping by next-generation sequencing of complementary DNA
Source: BMC Genomics. 2017 Nov 28;18:914. doi: 10.1186/s12864-017-4300-7 (PMC5704545; doi:10.1186/s12864-017-4300-7)
Supplement: Supplementary file 2 — Examples of alignment with BLAT. (PPT 70 kb) [file 12864_2017_4300_MOESM2_ESM.ppt]

## Slide 1
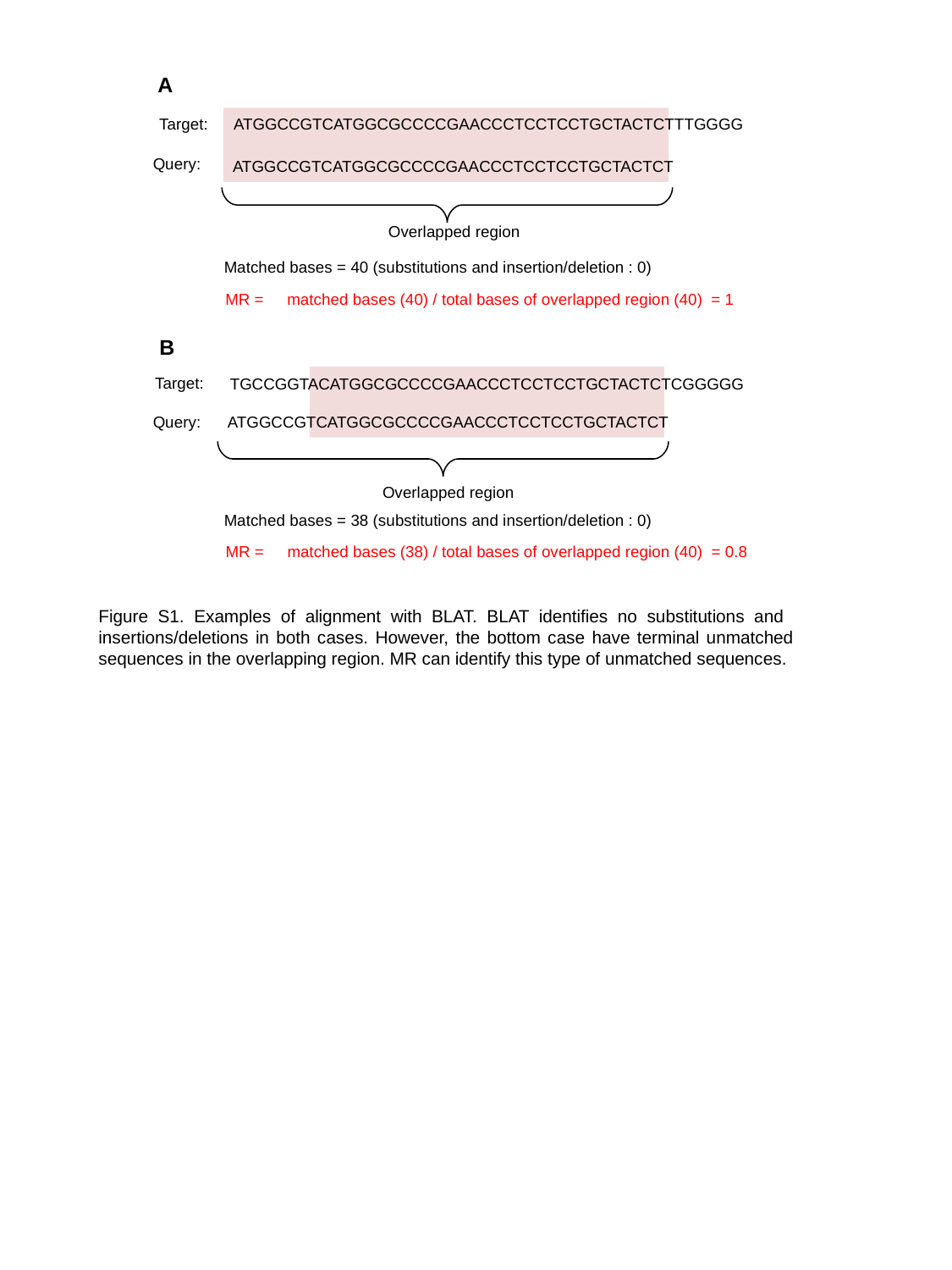

A
 Target:
ATGGCCGTCATGGCGCCCCGAACCCTCCTCCTGCTACTCTTTGGGG
Query:
ATGGCCGTCATGGCGCCCCGAACCCTCCTCCTGCTACTCT
Overlapped region
Matched bases = 40 (substitutions and insertion/deletion : 0)
MR =　matched bases (40) / total bases of overlapped region (40) = 1
B
 Target:
TGCCGGTACATGGCGCCCCGAACCCTCCTCCTGCTACTCTCGGGGG
ATGGCCGTCATGGCGCCCCGAACCCTCCTCCTGCTACTCT
Query:
Overlapped region
Matched bases = 38 (substitutions and insertion/deletion : 0)
MR =　matched bases (38) / total bases of overlapped region (40) = 0.8
Figure S1. Examples of alignment with BLAT. BLAT identifies no substitutions and insertions/deletions in both cases. However, the bottom case have terminal unmatched sequences in the overlapping region. MR can identify this type of unmatched sequences.
