# Supplementary material for: HLA genotyping by next-generation sequencing of complementary DNA
Source: BMC Genomics. 2017 Nov 28;18:914. doi: 10.1186/s12864-017-4300-7 (PMC5704545; doi:10.1186/s12864-017-4300-7)
Supplement: Supplementary file 4 — Calculation of the OL (Overlapped Length) between sequences of “Haplotype candidates” and “HLA cDNA database”. (PPT 174 kb) [file 12864_2017_4300_MOESM4_ESM.ppt]

## Slide 1
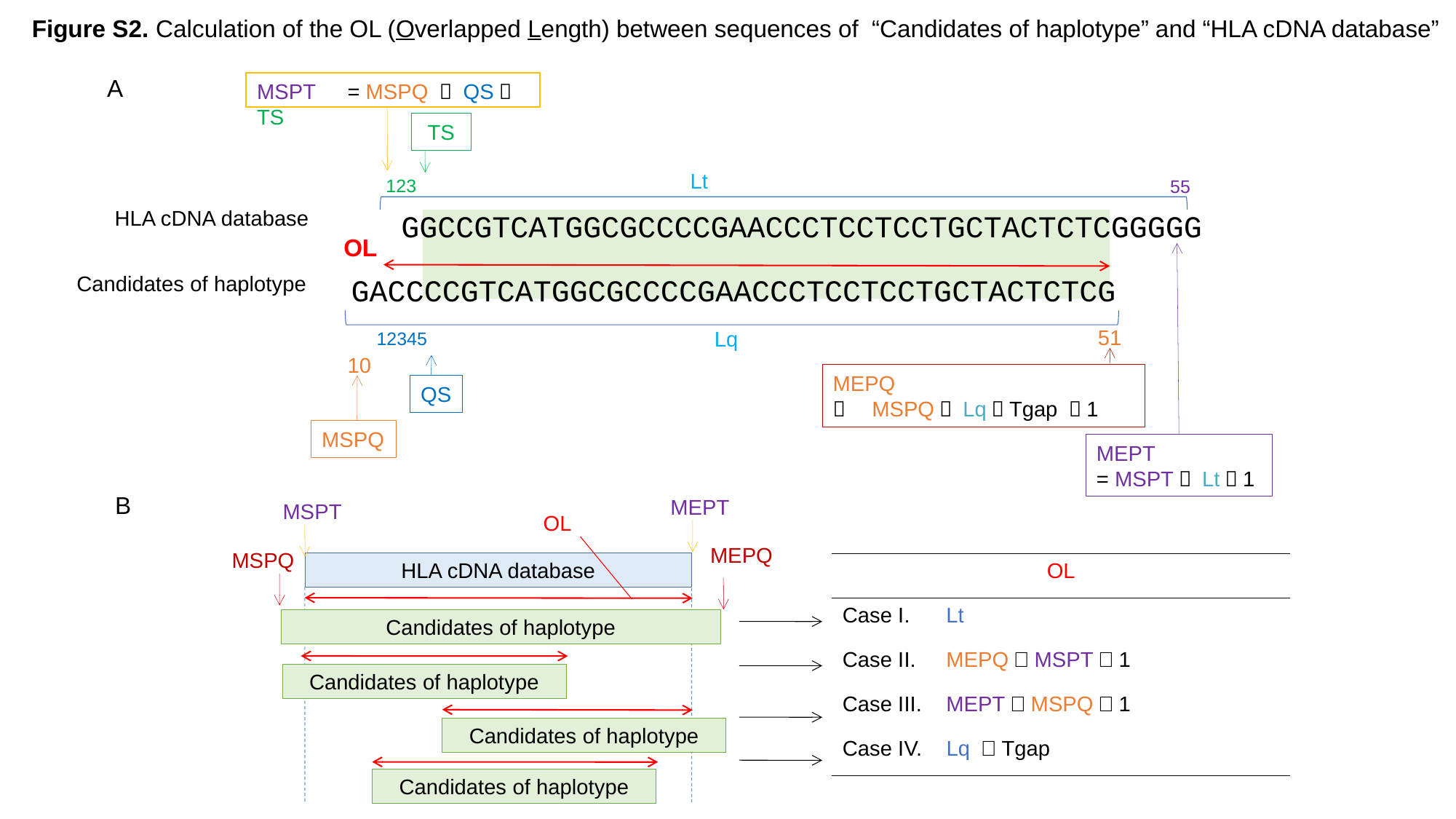

Figure S2. Calculation of the OL (Overlapped Length) between sequences of “Candidates of haplotype” and “HLA cDNA database”
A
MSPT　= MSPQ ＋ QS－ TS
TS
123
Lt
55
GGCCGTCATGGCGCCCCGAACCCTCCTCCTGCTACTCTCGGGGG
GACCCCGTCATGGCGCCCCGAACCCTCCTCCTGCTACTCTCG
12345
10
QS
MSPQ
MEPQ
＝　MSPQ＋ Lq＋Tgap －1
MEPT
= MSPT＋ Lt－1
51
Lq
HLA cDNA database
Candidates of haplotype
OL
B
MEPT
MSPT
MEPQ
MSPQ
HLA cDNA database
Candidates of haplotype
Candidates of haplotype
Candidates of haplotype
Candidates of haplotype
OL
| OL |
| --- |
| Case I. Lt |
| Case II. MEPQ－MSPT＋1 |
| Case III. MEPT－MSPQ＋1 |
| Case IV. Lq ＋Tgap |
