# Supplementary material for: HLA genotyping by next-generation sequencing of complementary DNA
Source: BMC Genomics. 2017 Nov 28;18:914. doi: 10.1186/s12864-017-4300-7 (PMC5704545; doi:10.1186/s12864-017-4300-7)
Supplement: Supplementary file 5 — Primers for measuring single-strand products by Real-time PCR. (PPT 139 kb) [file 12864_2017_4300_MOESM5_ESM.ppt]

## Slide 1
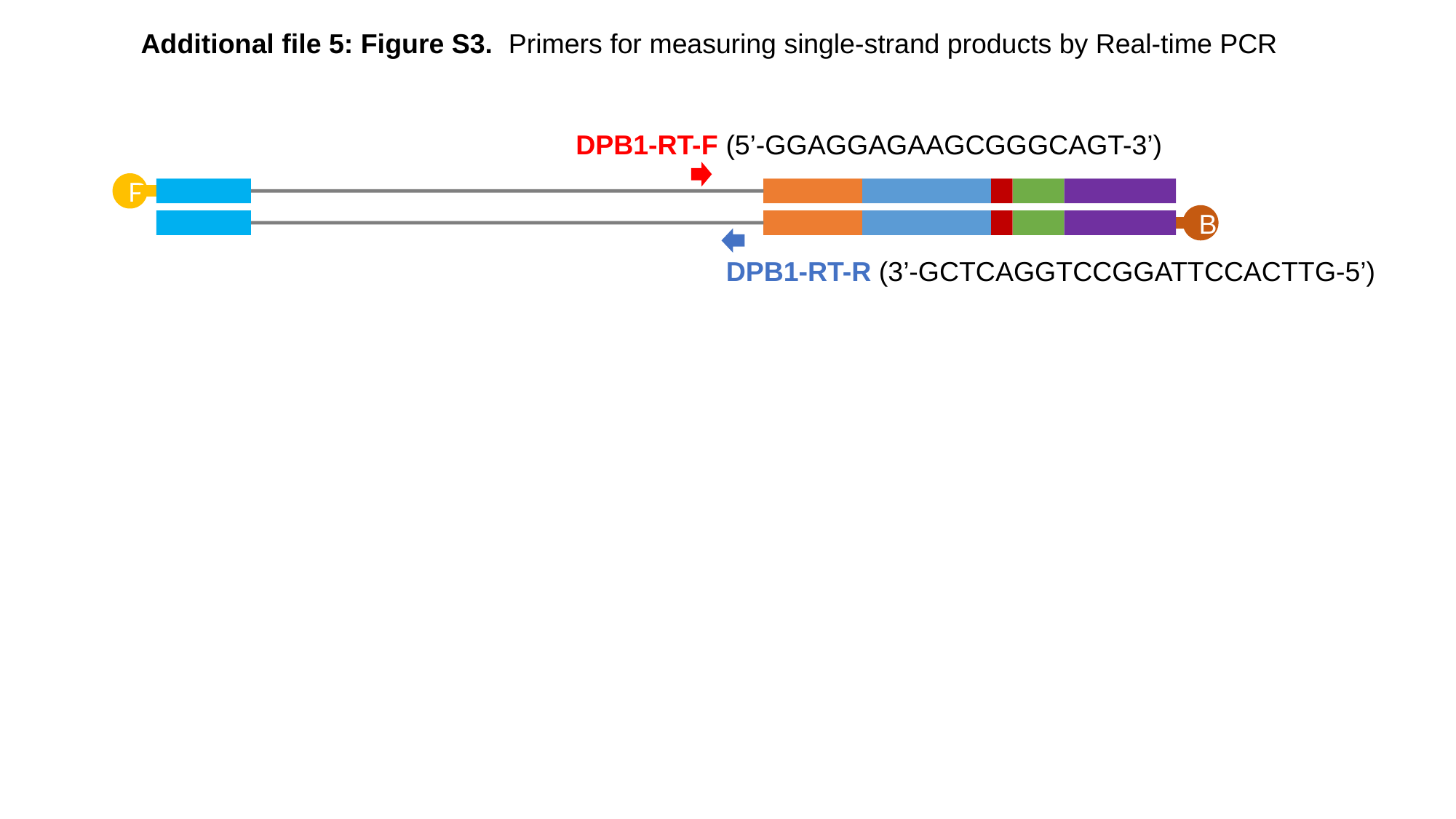

Additional file 5: Figure S3. Primers for measuring single-strand products by Real-time PCR
DPB1-RT-F (5’-GGAGGAGAAGCGGGCAGT-3’)
P
B
DPB1-RT-R (3’-GCTCAGGTCCGGATTCCACTTG-5’)
